# Investigating the Influence of MoS2 Nanosheets on *E*. *coli* from Metabolomics Level

**DOI:** 10.1371/journal.pone.0167245

**Published:** 2016-12-01

**Authors:** Na Wu, Yadong Yu, Tao Li, Xiaojun Ji, Ling Jiang, Jiajun Zong, He Huang

**Affiliations:** 1 College of Biotechnology and Pharmaceutical Engineering, Nanjing Tech University, Nanjing, China; 2 Jiangsu National Synergetic Innovation Center for Advanced Materials (SICAM), Nanjing, China; 3 College of food science and light industry, Nanjing Tech University, Nanjing, China; 4 School of Pharmaceutical Sciences, Nanjing Tech University, Nanjing, China; Institute of Materials Science, GERMANY

## Abstract

Molybdenum disulfide, a type of two-dimensional layered material with unique properties, has been widely used in many fields. However, an exact understanding of its toxicity remains elusive, let alone its effects on the environmental microbial community. In this study, we utilized metabolomics technology to explore the effects of different concentrations of molybdenum disulfide nanosheets on *Escherichia coli* for the first time. The results showed that with increasing concentration of molybdenum disulfide nanosheets, the survival rate of *Escherichia coli* was decreased and the release of lactic dehydrogenase was increased. At the same time, intracellular concentrations of reactive oxygen species were dramatically increased. In addition, metabolomics analysis showed that high concentrations of molybdenum disulfide nanosheets (100, 1000 μg/mL) could significantly affect the metabolic profile of *Escherichia coli*, including glycine, serine and threonine metabolism, protein biosynthesis, urea cycle and pyruvate metabolism. These results will be beneficial for molybdenum disulfide toxicity assessment and further applications.

## Introduction

The environmental safety assessment of nanomaterials has long been investigated by many scientists and a number of studies have been conducted to investigate the effects of nanomaterials on environmental microorganisms. For instance, Lyon *et al*. have shown that C60 in powder form had no impact on bacteria while an aqueous suspension of C60 generated toxic effects [1]. Li et al. have also demonstrated that carbon nanotubes showed antimicrobial activity on the studied bacterial strains [2]. Some researchers found that graphene could damage the cell membrane of *Escherichia coli* (*E*. *coli*) and thereby, showed strong antibacterial activity [3,4].

Molybdenum disulfide (MoS_2_) has a graphene-like structure, which is a typical layered crystal, and consists of sulfur (S) and molybdenum (Mo) atoms, with individual layers bound to each other by van der Waals forces [5]. MoS_2_ possesses excellent biocompatibility, strong visible light absorption, fluorescence quenching characteristics and a number of other interesting properties [6–8]. Due to these distinct electronic and physical/chemical properties, MoS_2_ has wide applications in many fields, such as opto-electronics, channel materials, biomedicine and dry lubrication [9–13]. Since ultrathin MoS_2_ shows much more attractive properties compared with bulk MoS_2_, large efforts have been made to develop methods for growing high quality ultrathin MoS_2_ films on various substrates. However, traditional top-down methods such as mechanical or chemical exfoliation are not suitable for the fabrication of large area, high-quality ultrathin MoS_2_ films [14–16]. Fortunately, MoS_2_ monolayers have been successfully grown on SiO_2_/Si substrates by chemical vapor deposition (CVD) [17]. Moreover, pulsed laser deposition (PLD) also showed great potential for growing monolayer and thin multi-layer MoS_2_ films [18].

Since MoS_2_ has a layered structure, the properties of MoS_2_ thin films are significantly linked to the number of their layers [19][20]. For example, the in-plane stiffness and breaking strength of monolayer MoS_2_ is higher than that of bulk MoS_2_ crystals [21]. When the number of the MoS_2_ layers increases, the inter-layer van der Waals forces within MoS_2_ start to significantly suppress vibrations, which is the main reason why bulk MoS_2_ has a higher restoring force than monolayer MoS_2_ [14]. Bulk MoS_2_ is a semiconductor material with an indirect band gap of 1.2 eV, and is often used as a photocatalyst, but also as a dry lubricant. A monolayer of MoS_2_, on the other hand, has a 1.9 eV direct band gap and possesses prominent electro- and photoluminescent properties [22]. Moreover, it is generally believed that the toxicity of 2D layered MoS_2_ depends on its defect density, exfoliation parameters and chemical composition [23]. Since the layer number can affect the surface area, defects and edge parameters of the MoS_2_ nanosheets, it has been found that the toxicity of MoS_2_ nanosheets increased with decreasing layer number [24].

Due to the increasingly broad application of MoS_2_, the opportunities for MoS_2_ to be released to the environment and thus come in contact with environmental microorganisms will likely only increase in the future. Therefore, assessing the effects of MoS_2_ on microbial communities has become increasingly urgent and some efforts have already been made towards this goal. For example, Nilam and co-workers discovered that the graphene-like molybdenum disulfide nanosheets (MSNs) possessed antibacterial properties on *E*. *coli* and *B*. *subtilis*, by measuring reactive oxygen species (ROS) and morphological observation [25]. Marek Kostecki *et al*. have demonstrated that MSNs have antibacterial and antifungal properties by SEM observation [26]. These preliminary experiments gave new insights useful for assessing the microbial toxicity of MoS_2_. However, an in-depth mechanistic explanation for how MoS_2_ affects microbial cells is still needed.

In fact, changes in microbial metabolites can directly reflect microbial responses to environmental stimuli. Metabolomics technology as a new set of tools has been widely used in the evaluation of the toxicity of nanomaterials [27]. For instance, Zhao et al. utilized gas chromatography-mass spectrometry (GC-MS) based on metabolomics to evaluate the toxicity of copper nanoparticles [28]. Ratnasekhar *et al*. used metabolomics to study the perturbations in the metabolome of *Caenorhabditis elegans* exposed to titanium dioxide nanoparticles [29].

Taken together, these observations have inspired the present work, in which gas chromatography-mass spectrometry (GC-MS), metabolomics technology and other molecular biology approaches have been employed to unveil the mechanism of how MoS_2_ nanosheets affect the important model bacterium *E*. *coli*.

## Materials and Methods

### MoS_2_ morphology observation and chemical element analysis

The MoS_2_ nanosheets used in this study were purchased from Sigma-Aldrich (99.995%, Sigma-Aldrich, St. Louis, USA). According to the method reported previously [26], field emission scanning electron microscopy combined with energy dispersive spectrometry (FE-SEM, Hitachi S-4800) was utilized to determine the surface morphology and relative abundance of chemical elements of the MoS_2_ samples. In addition, the MoS_2_ samples were analyzed using a HR800 Raman Microscope (Horiba Jobin Yvon, France) and focalized using a 40 × objective with an excitation wavelength of 514 nm, adjusting the exposure time to acquire the correct spectrum.

### Strain, cultivation and fermentation

We used *E*. *coli* MG1655 as a model bacterium to evaluate the antibacterial activity of MoS_2_ nanosheets (MSNs), and the strain was kindly provided by the group of Prof. Guoqiang Chen (School of Life Sciences, Tsinghua University, China). *E*. *coli* was maintained on Luria Bertani (LB) liquid medium containing 10 g/L tryptone (OXIOD, UK), 5 g/L yeast extract (OXIOD, UK) and 5 g/L NaCl, with pH set to 7.0, at 37°C under constant orbital shaking at 220 rpm for up to 12 h. The LB liquid medium was supplemented with MSNs at concentrations of 0, 1, 10, 100 and 1000 μg/mL. For fermentation, 5% v/v aliquots of seed culture were used to inoculate 50 mL fermentation media containing 10 g/L tryptone, 5 g/L yeast extract, 10 g/L NaCl and 15 g/L glucose, which were subsequently incubated at 37°C under constant orbital shaking at 220 rpm for a further 12 h.

### Recording of growth curves

*E*. *coli* was cultured in LB media with 0, 1, 10, 100, or 1000 μg/mL of MSNs. Sampling was carried out at 2 h intervals during the course of 12 h of fermentation. Cell growth was determined by measuring the optical density at 600 nm (Lambda-25 spectrophotometer, Perkin-Elmer, USA) in six parallel measurements for each time-point.

### Determination of viability

Methylthiazolyldiphenyl-tetrazolium bromide (MTT) reagent (Sigma-Aldrich, USA) was used for cell viability measurements. *E*. *coli* was exposed to 0, 1, 10, 100, or 1000 μg/mL of MSNs in PBS (pH 7.0). Subsequently, the cells were incubated in PBS at 37°C under constant orbital shaking at 220 rpm for 6 h, after which 20 μL PBS solution containing cells, 20 μL MTT solution (1 mg/mL) and 60 μL fresh PBS were transferred into an Eppendorf tube and incubated in a thermostat water bath for 30 min at 37°C. Subsequently, the tube was centrifuged at 10000 g for 5 min and the supernatant removed. 300 μL dimethyl sulfoxide (DMSO) was added to each tube to dissolve the sediment and the tubes were centrifuged at 10000 g for another 5 min. The supernatants were transferred to 96-well plates and the absorbance at 490 nm was measured using a plate-reader (Molecular Devices, SpectraMax M3, USA). The results are expressed as the means ± SD of six parallel measurements.

### Determination of membrane stability

*E*. *coli* was exposed to 0, 1, 10, 100, or 1000 μg/mL of MSNs in PBS (pH 7.0). Subsequently, the cells were incubated in PBS at 37°C under constant orbital shaking at 220 rpm for 6 h, after which the cells were separated by centrifugation at 4000 rpm for 5 min and the supernatants transferred into 96-well plates. Lactate dehydrogenase (LDH) activity was determined using a lactate dehydrogenase kit (Sigma-Aldrich, USA), according to the manufacturer’s protocol, with final detection of absorption at 450 nm using a Lambda-25 plate reader (Perkin-Elmer, USA). The results are expressed as the means ± SD of six parallel measurements.

### Determination of oxidative stress

2,7-Dichlorodihydrofluorescein diacetate (DCFH-DA) (Sigma-Aldrich, USA) was added to 50 mL LB media as a 10mM stock in DMSO, and incubated at 37°C under constant orbital shaking at 220 rpm for 20 min, after which the cells were washed twice with PBS and dispersed in PBS solutions containing 0, 1, 10, 100, or 1000 μg/mL of MSNs. Subsequently, the PBS solutions containing the cells and the MSNs were incubated at 37°C under shaking at 220 rpm for another 4 h, after which the PBS supernatants were transferred into the wells of a 96-well plate and evaluated on a fluorescent spectrophotometer (SpectraMax M3, Molecular Devices, USA) using an excitation wavelength of 488 nm and an emission wavelength of 525 nm. The results are expressed as the means ± SD of six parallel measurements.

### Transmission electron microscopy

After 12 h of incubation at 37°C under shaking at 220 rpm, the cells were collected by centrifugation at 8000 rpm and 5°C for 5 min. The pellets were re-suspended in 25% glutaraldehyde and fixed for 12 h, after which the cells were washed with PBS three times. 2% Osmium tetroxide (OsO_4_) was added to the pellets and contacted for 1 h on a sample rotator. The cells were dewatered by subsequent rinses with solutions comprising 35%, 50%, 70%, 90% and 95% ethanol, followed by a final rinse in absolute ethanol. After contacting with propylene oxide for 30 min, propylene oxide/resin (Meryer, China) (1:1) was added and allowed to react for 12 h at 45°C. Finally, the cells were embedded in 100% resin and polymerization was conducted in an oven at 70°C for 24 h, after which the samples were prepared as 90 nm sections using an EM UC6 Ultramicrotome (Leica, Germany). After staining with 3% uranyl acetate and lead citrate (Beijing chemical works, China) for double staining, the sections were examined under a JEM-1011 transmission electron microscope (JEOL, Japan).

### GC-MS metabolomic experiments

Media samples were collected by centrifugation at 5000 rpm for 8 min at -20°C. The supernatant was removed and 1.5 mL of ice-cold (-20°C) 60% methanol was added to the pellets to terminate metabolic activity. The mixtures were transferred into 2 mL pre-weighted Eppendorf tubes, and each tube frozen and thawed five times in liquid nitrogen and subsequently centrifuged at 15000 rpm and -20°C for 15 min. The supernatants were stored at -80°C. The remaining materials were suspended in 1 mL of ice-cold (-20°C) 60% methanol in water and centrifuged same as above. Subsequently, the resulting supernatant was mixed with the first supernatant and carefully transferred into a 1.5 mL Eppendorf tube, after which 10 μL of a 0.2 mg/mL ribitol (Sigma-Aldrich, USA) solution was added as internal standard and the samples dried under nitrogen gas. The dried samples were mixed with 50 μL methoxylamine hydrochloride /pyridine (20 mg/mL) (Sigma-Aldrich, USA) and incubated in a water bath at 37°C for 80 min. The compounds were blended with 80 μL MSTFA (N-Methyl-N-(trimethylsilyl) trifluoroacetamide) (Sigma-Aldrich, USA) and incubated in a water bath at 37°C for 80 min, after which the samples were centrifuged at 10000 g for 5 min and a 100 μL aliquot of the supernatant transferred to a fresh Eppendorf tube and used directly for GC-TOF/MS detection on a gas chromatograph-mass spectrometer (Trace GC2000 DSQ, Agilent, USA).

### Bioinformatics analysis

AMDIS software (NIST, v2.69, Gaithersburg, MD, USA) coupled with MSD Chemstation software (Agilent Technologies, G1701 EAE.02.00.493, USA) was used for the qualitative and quantitative analysis of mass spectrometry data. MSD software was used to integrate the peak areas. Each peak was qualitatively analyzed in the chromatograms. The spectral libraries used mainly contained data from NIST 2005 and Wiley. All peak areas were normalized for further data processing and imported into Expander (version 6.0) software for cluster analysis. According to a method reported earlier [30], metabolite data displaying significant changes were imported into MetaboAnalyst3.0 for enrichment analysis and pathway analysis. Pathways with an impact value >0.2 were considered to be significantly affected.

## Results and Discussion

### Characterization of MoS_2_ nanosheets

**Fig 1:**
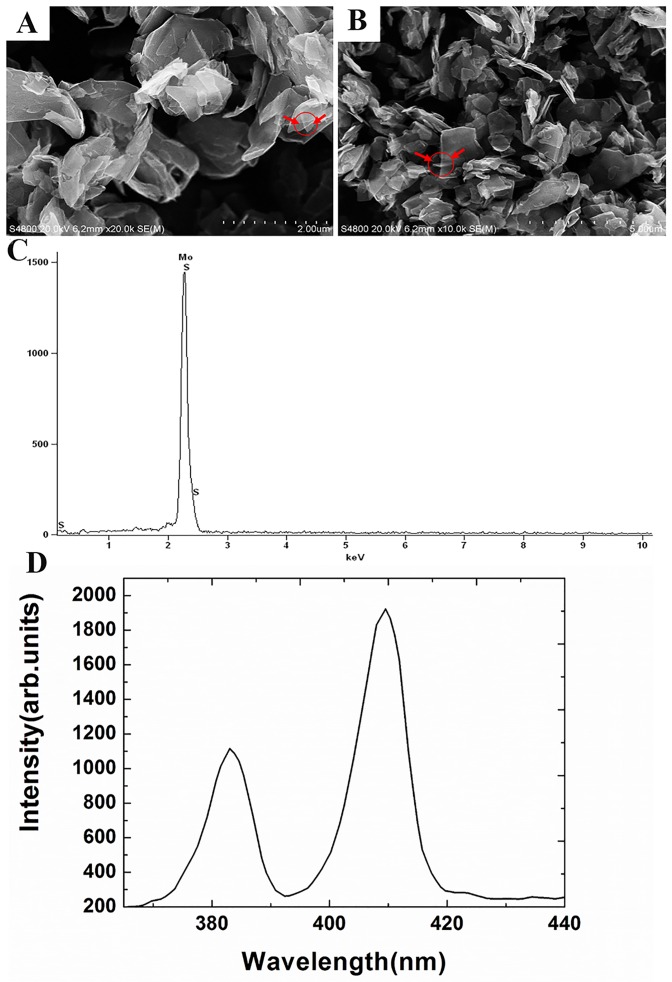
(A-B) Scanning electron microscopy (SEM) image of the molybdenum disulfide nanosheets (MSNs); (C) EDS spectra for determining the chemical composition of the MSNs; (D) Raman spectrum of the MSNs.

As shown in Fig 1A and 1B, MoS_2_ nanosheets **(**MSNs) were found to present as flower-like flakes that showed a layered crystal structure. It has been reported that MSNs seemed to become transparent when the layer number decreased, since the MSNs became thin enough for light to pass through [18,31]. Herein, we also found that some parts of the bulk MSNs looked transparent (Fig 1A and 1B, indicated with red circles and arrows), since some of the MSN flakes were very thin. We further characterized the chemical components of the MSNs we used. The EDS results indicated that the MoS_2_ samples only contained sulfur and molybdenum atoms without any significant impurities (see Fig 1C and S1 Table). Raman spectroscopy has been widely applied to characterize the structural properties of MSNs and it has been possible to determine the number of layers, as the vibrational spectrum is sensitive to sample thickness and by inference, to the number of layers [18]. In our work, the MSNs were thus further characterized by Raman spectroscopy (Fig 1D). As shown in Fig 1D, the Raman spectrum of the MSNs had two prominent peaks (383 cm^-1^ and 409 cm^-1^), corresponding to the in-plane (E^1^_2g_) mode and the out-of-plane (A_1g_) mode, respectively [32]. The spacing between the E^1^_2g_ and A_1g_ modes was about 26 cm^-1^, which suggested that the used MSNs were composed of bulk MoS_2_ [14, 33].

### The effect of MSNs on the growth, viability, LDH release and ROS accumulation of *E*. *coli*

**Fig 2:**
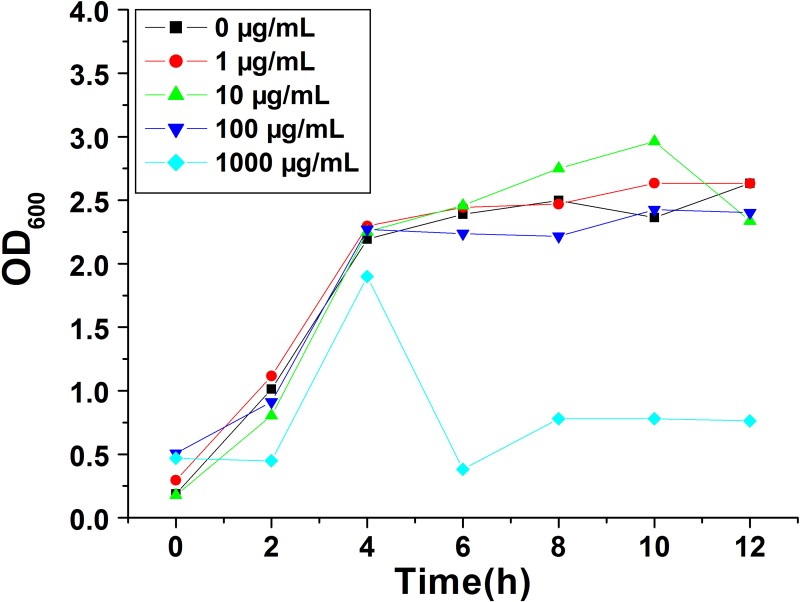
The growth curves of *E*. *coli* cultured in LB broth with the addition of different concentrations of MSNs. black rectangles: 0, red circles: 1, green triangles: 10, blue inverted triangles: 100 and cyan diamonds: 1000 μg/mL of MSNs.

To explore the influence of MSNs on the growth of *E*. *coli*, cells were incubated in broth with a series of MSNs concentrations (0, 1, 10, 100, 1000 μg/mL). As shown in Fig 2, the OD values of cultures grown in broth with the addition of 1, 10 or 100 μg/mL of MSNs seemed similar to that of the control. However, the growth curves of *E*. *coli* became unstable when the concentration of MSNs increased to 1000 μg/mL. Hence, these results indicated that MSNs with a concentration of no more than 100 μg/mL had no remarkable impact on the growth of *E*. *coli*. However, when 1000 μg/mL of MSNs was added to the broth, OD values were lower than the other groups and decreased significantly after 4 h, which indicated that a high concentration of MSNs could significantly inhibit the growth of *E*. *coli*.

**Fig 3:**
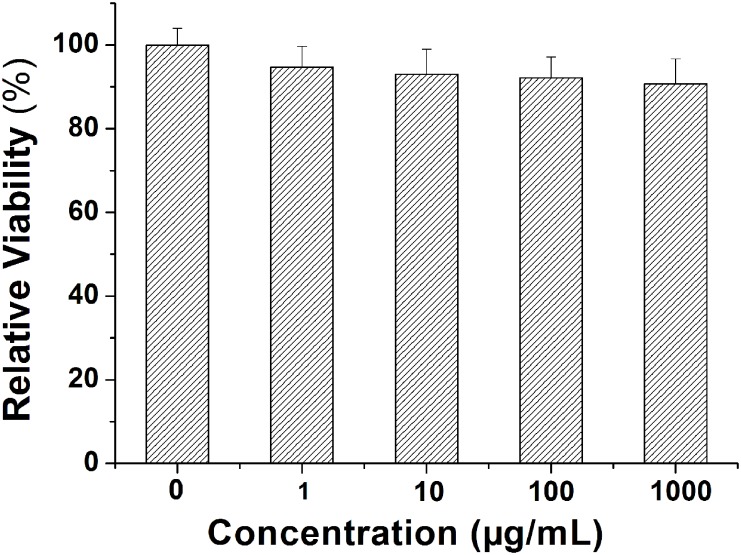
Relative viability *E*. *coli* cells exposed to different concentrations (0, 1, 0, 100, 1000 μg/mL) of MSNs.

We further investigated the viability of *E*. *coli* cells exposed to different concentrations of MSNs. Fig 3 illustrates that the experimental groups contacted with different dosages of MSNs showed a slight and dose-dependent inhibition of viability. These findings were consistent with other published work, which reported that MoS_2_ showed mild direct cytotoxicity [34,35]. In our experiment, *E*. *coli* was cultured in aqueous solution in shake flasks and we hypothesized that the low observed toxicity might be attributed to the very low solubility of MSNs.

Nano-materials can be damaging to cells, mainly due to damage to cell membranes and the induction of oxidative stress as discussed in previous reports [36,37]. The excessive accumulation of reactive oxygen species (ROS) can damage the cell membrane and reduce its stability. In this regard, lactate dehydrogenase (LDH) is a vital indicator which can be used as a proxy for oxidative damage to the cell membrane [38]. Therefore, we evaluated LDH release and ROS accumulation to investigate the impact of MSNs on cell membrane stability in *E*. *coli*.

**Fig 4:**
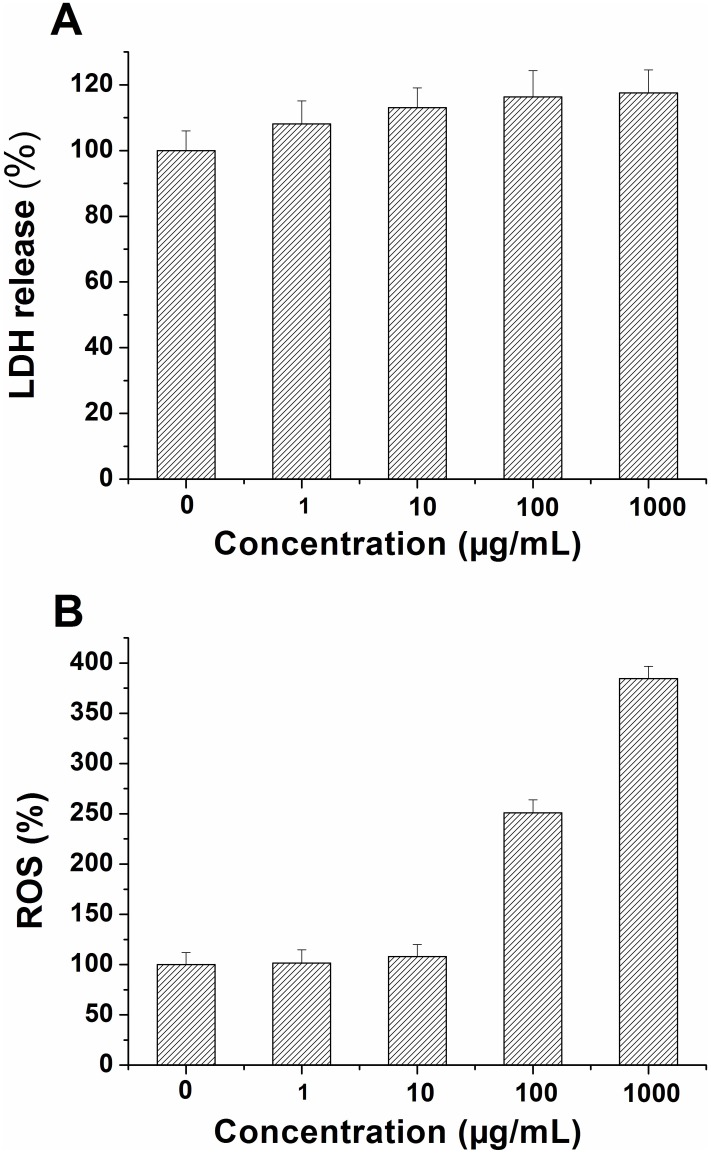
(A) Relative lactate dehydrogenase (LDH) release from *E*. *coli* exposed to different concentrations (0, 1, 0, 100, 1000 μg/mL) of MSNs; (B) Relative intercellular reactive oxygen species (ROS) levels of *E*. *coli* exposed to different concentrations (0, 1, 0, 100, 1000 μg/mL) of MSNs.

Our experimental observations showed that when *E*. *coli* was exposed to MSNs, LDH release increased compared to the control. Fig 4A shows that the LDH release was increased by 8.1%, 13.1%, 16.3% and 17.6% at MSNs concentrations of 1, 10, 100 and 1000 μg/mL, respectively. Tu *et al*. used computer simulation to model the impairment of cell membranes caused by graphene nanosheets. They demonstrated that both graphene nanosheets and graphene oxide nanosheets could insert into *E*. *coli* membranes. This phenomenon explains how graphene nanosheets and graphene oxide nanosheets could generate obvious membrane stress and reduce cell viability of *E*. *coli* [39]. The increase of LDH release may be caused by interactions between the MSNs and the surface of the *E*. *coli* cell membrane, which in turn might reduce membrane stability and the resistance of *E*. *coli* to external substances. MSNs can penetrate cell walls and expose the cell membrane, which decreases its stability. Finally, the amount of LDH apparently increased in this bacterium.

It is well known that ROS are generated on the surface of nano-materials, which further induces the cells to generate more ROS [40,41]. ROS generation is a cumulative process, whereby only reaching a certain value of damage can cause significant permanent intracellular oxidative damage. We further investigated the influence of MSNs on intracellular ROS after exposing *E*. *coli* to different doses of MSNs. As shown in Fig 4B, when the concentrations of MSNs were in the range of 1–100 μg/mL, the levels of ROS in *E*. *coli* increased only slightly. On the other hand, MSN concentrations in the range of 100–1000 μg/mL caused the generation of significant amounts of ROS. These results indicated that MSNs, just like other nanomaterials such as fullerene, graphene and its derivatives, could cause an increase of intracellular ROS, which might finally cause damage to the cells [42–44]. Nel et al. did a considerable amount of work on the toxicity of nanomaterials and found that the generation of ROS might be associated with surface defects, electron—hole pair generation, chemical dissolution and release of toxic metal ions from nanomaterials [36]. Judging by the SEM images (Fig 1D), we also found that the MSNs possessed surface defects, and we thus speculated that MSNs could catalyze ROS formation due to their discrete crystal planes and surface defects, which can result in the production of active electrons on their surface. The next reaction step might be that the excited electrons turned oxygen molecules into superoxide anions (O_2_^.-^), which eventually generated further ROS via disproportionation [41].

### The effects of MSNs on the ultrastructure of *E*. *coli*

**Fig 5:**
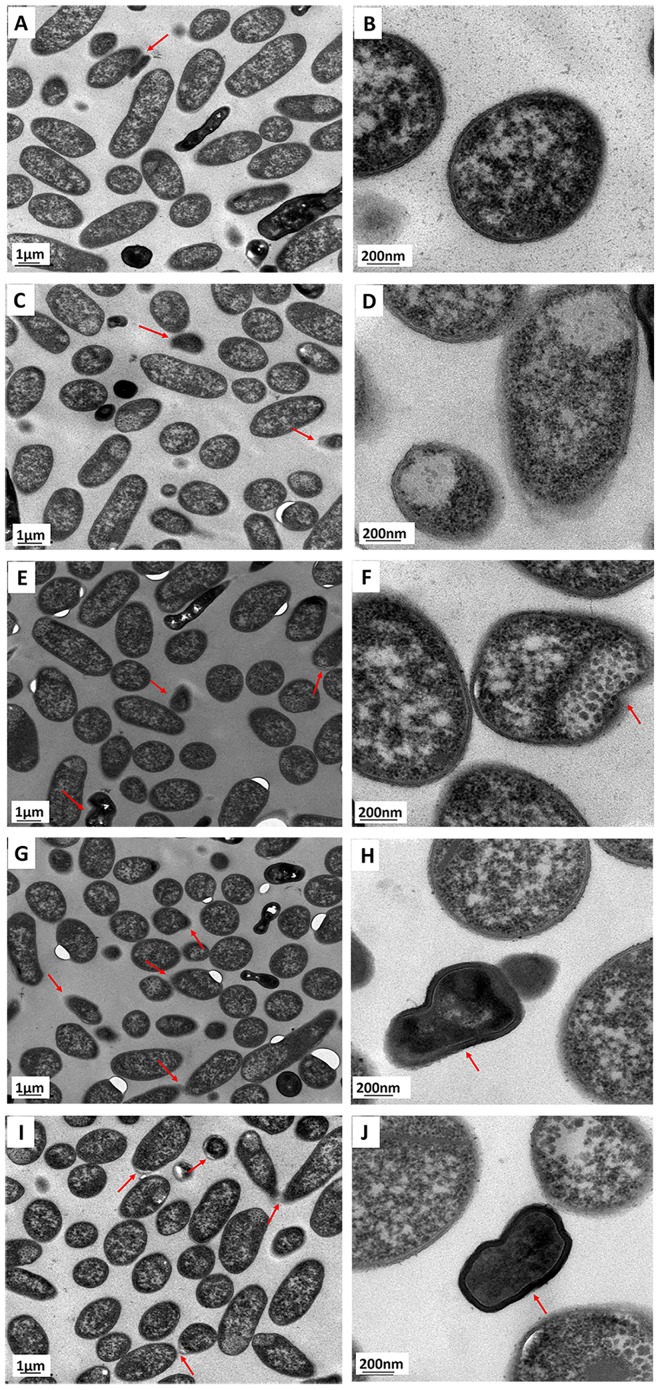
Subcellular structures of *E*. *coli* cells that were exposed to 0 μg/mL (A-B), 1 μg/mL (C-D), 10 μg/mL (E-F), 100 μg/mL (G-H) and 1000 μg/mL (I-J) of MSNs, respectively, for 12 h. The observed shrunken *E*. *coli* cells are marked with red arrows.

**Fig 6:**
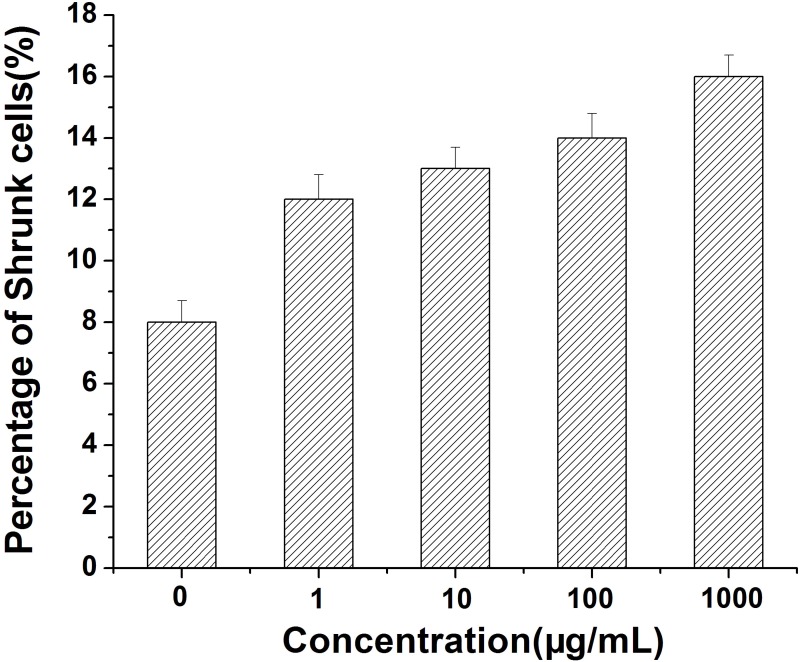
The percentage of shrunken *E*. *coli* cells in cultures after exposure to the indicated concentrations (0, 1, 10, 100, 1000 μg/mL) of MSNs.

When *E*. *coli* was cultured without the addition of MSNs, the cells showed a long and tubular form, and the cell membrane was relatively intact. Some of the cells were found to be shrunken (see Fig 5A and 5B). When *E*. *coli* was exposed to low doses of MSNs (1, 10 μg/mL), some cells that changed in cell structure were found (Fig 5C–5F). On the other hand, when *E*. *coli* was exposed to high doses of MSNs (100, 1000 μg/mL), some of the cells were also found to be shrunk and their cell membranes appeared to be broken (Fig 5G–5J). These results suggested that MSNs affected the cellular structure of *E*. *coli* in a dose-depended manner, which was similar to the observations reported for other nanomaterials [45,46]. In order to quantify the TEM results, the percentage of shrunken cells was calculated by counting the shrunken *E*. *coli* cells in the TEM images. We found that the percentages of shrunken cells were 8%, 12%, 13%, 14% and 16% when the *E*. *coli* were treated with 0, 1, 10, 100 and 1000 μg/mL of MSNs, respectively (Fig 6). It was thus found that the percentage of shrunken cells increased slightly when the concentration of MSNs was raised from 0 μg/mL to 1000 μg/mL. This result was in accordance with the results of LDH release and ROS analysis (Fig 4A and 4B). We hypothesized that high doses of MSNs damaged the cellular structure of *E*. *coli*, which caused increased LDH release. Moreover, high concentrations of MSNs stimulated the production of high levels of ROS, which would in turn further damage the cellular components.

### The effects of MSNs on the metabolism of *E*. *coli*

Metabolomics, a rapidly developing technology, has been used to investigate the physiological and metabolic states of cells in a comprehensive manner. Herein, we utilized metabolomics methods to evaluate the effects and toxicological mechanisms of MSNs.

### Cluster analysis

**Fig 7:**
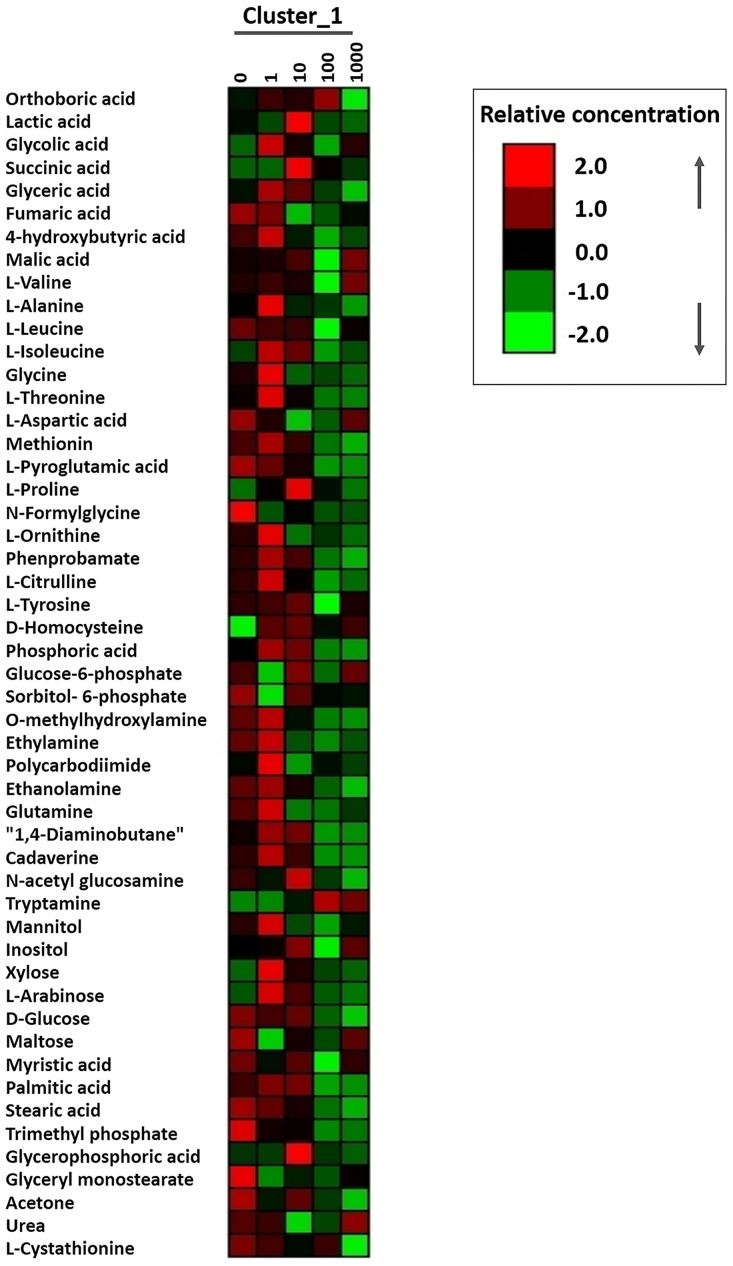
Heat map representation of the metabolites in the *E*. *coli* cells that were exposed to the indicated concentrations (0, 1, 10, 100, 1000 μg/mL) of MSNs. The up-regulated and down-regulated metabolites were shown in red and green, respectively.

Using GC-MS, we evaluated the differences in the metabolic profiles of *E*. *coli* cells exposed to different concentrations of MSNs relative to the untreated control group. A total of 51 metabolites were filtered out, including amino acids, esters, organic acids, sugars, alcohols, amines, long chain fatty acids and phosphate compounds (Fig 7).

As shown in Fig 7, various metabolites were influenced by exposure of the cells to MSNs. Interestingly, most of these metabolic changes were already induced by low concentrations of MSNs (1, 10 μg/mL) while they were suppressed by high concentrations (100, 1000 μg/mL). For instance, the contents of amino acids, phosphoric acid, and tricarboxylic acid cycle-related metabolites increased or decreased when *E*. *coli* was exposed to low (1, 10 μg/mL), or high (100, 1000 μg/mL) concentrations of MSNs, respectively.

The amino acid metabolism is particularly sensitive to environmental disturbances. Li et al. reported that environmental disturbances to microorganisms might result in increased levels of intracellular amino acids caused by the degradation of abnormal, misfolded proteins. Afterwards, the microorganisms would re-synthesize correctly folded proteins to maintain their normal functions in adverse circumstances. However, if adverse circumstances exerted a negative pressure so severe that the microbes could not withstand them, the cells would stop synthesizing proteins and the concentration of intracellular amino acids would decrease [47]. Phosphoric acid on the other hand, is not only a vital component of the phospholipid bilayer, but also acts as an activator for a number of protein kinases that regulate important signal transduction pathways and make the cells induce stress responses against adverse environmental conditions [48]. Therefore, the changes in the content of amino acids and free phosphoric acid shown in Fig 7 might be caused by stress mechanisms intrinsic to the microorganism. When exposed to low concentrations of MSNs, *E*. *coli* would resist adverse environmental conditions by producing more amino acids and accumulating free phosphoric acid, whereas high dosages of MSNs, which might exceed the tolerance range of *E*. *coli*, would result in degradation of amino acids and decrease of free phosphoric acid.

The tricarboxylic acid (TCA) cycle, also known as the citric acid cycle, is a series of enzyme-catalyzed chemical reactions which occur in many bacteria. The contents of tricarboxylic acid cycle-related metabolites (e.g. lactate, 4-hydroxybutyrate, succinate, glycine) showed a similar change to the changes of amino acids. This result suggested that the metabolic fluxes in the TCA cycle were increased when *E*. *coli* was exposed to low concentrations of MSNs, but high dosages of MSNs suppressed the TCA cycle. The levels of metabolites related to the TCA cycle are also decreased under different stress conditions in *E*. *coli* [49].

### Enrichment analysis

**Fig 8:**
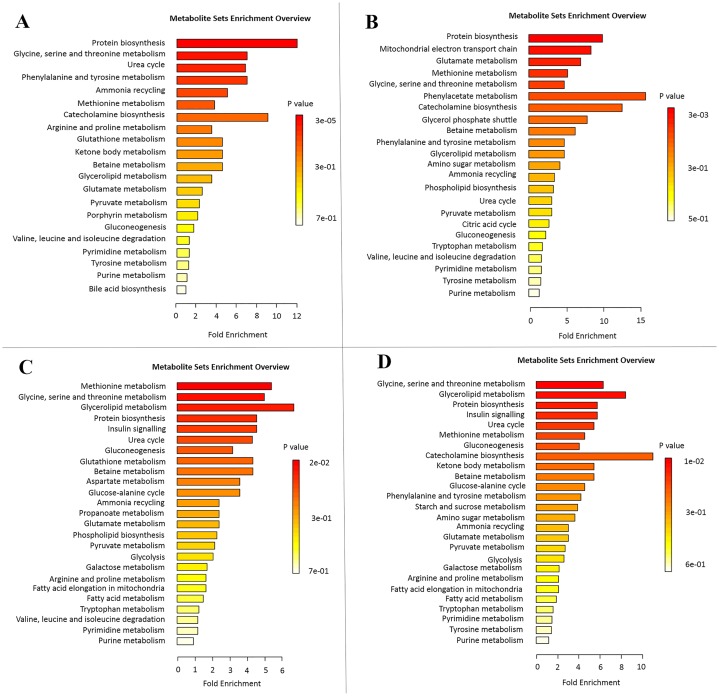
Enrichment analysis of the metabolites in the *E*. *coli* cells that were exposed to 1 μg/mL (A), 10 μg/mL (B), 100 μg/mL (C) and 1000 μg/mL (D) of MSNs.

As shown in Fig 8, when *E*. *coli* was exposed to different concentrations of MSNs, the perturbed metabolites were mainly concerned with protein biosynthesis, glycine, serine and threonine metabolism, the urea cycle, phenylalanine and tyrosine metabolism, ammonia recycling, methionine metabolism, and the electron transport chain. Interestingly, four functions, namely protein biosynthesis, glycine, serine and threonine metabolism, urea cycle, and methionine metabolism, were significantly changed in all the experimental groups. These results suggested that MSNs mainly affect protein biosynthesis and the amino acid metabolism of *E*. *coli*.

### Pathway analysis

**Fig 9:**
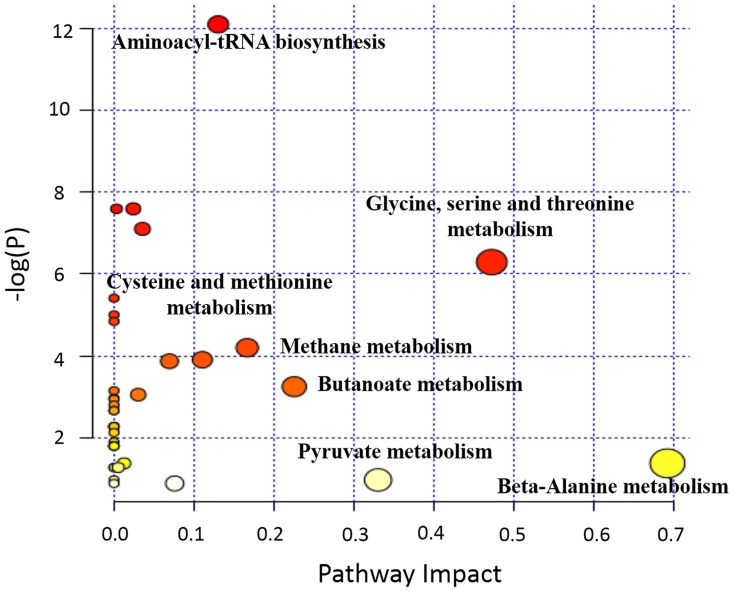
Pathway analysis of the metabolites in the *E*. *coli* cells that were exposed to 1000 μg/mL of MSNs.

MetPA network tools were utilized to further analyze the effects of different concentrations of MSNs on the metabolic pathways of *E*. *coli*. As shown in Fig 9, we found that glycine, serine and threonine metabolism, as well as beta-alanine metabolism, but also the metabolisms of carboxylic acids such as pyruvate and butanoate were all significantly affected by exposure to 1000 μg/mL of MSNs. The pathway analysis results were almost the same as in the ones shown in Fig 9, wherein *E*. *coli* was exposed to 1 μg/mL, 10 μg/mL, or 100 μg/mL (S1, S2 and S3 Figs). These results confirmed that MSNs could indeed significantly affect the amino acid-related metabolic pathways. As discussed above, due to stress response mechanisms found in many microorganisms, *E*. *coli* might modulate amino acid- or protein-related metabolic pathways to adapt to the adverse environment.

Pyruvate can enter the TCA cycle and serve as a key metabolite for the microbes to maintain normal physiological functions [50]. Wang *et al*. found that hexavalent chromium exposure suppressed pyruvate metabolism in *Shewanella oneidensis* [51]. In agreement with this finding, we also found that MSNs significantly affected the pyruvate metabolism, suggesting that MSNs might hamper the normal functions of *E*. *coli* by disturbing this important metabolite.

## Conclusions

Herein, we used different methods, including metabolomics, to systematically investigate the influence of MSNs on *E*. *coli*. Our experimental results showed that high concentrations (100 μg/mL and more) of MSNs caused damage to cell membranes, induced ROS accumulation, and reduced viability. Exposure to low concentrations of MSNs (1, 10 μg/mL) on the other hand, increased the intracellular concentrations of many metabolites in *E*. *coli*. Interestingly, exposure to high concentrations of MSNs (100, 1000 μg/mL), conversely, lowered the concentrations of these same metabolites. Metabolomics analysis further revealed that exposure to high concentrations of MSNs could significantly affect several metabolic pathways such as amino acid related metabolism and pyruvate metabolism. These findings provide new insights for assessing MoS_2_ microbial toxicity.

## Supporting Information

S1 Fig Pathway analysis of perturbed metabolites in *E*. *coli* cells exposed to 1 μg/mL MSNs. (TIF)

S2 Fig Pathway analysis of perturbed metabolites in *E*. *coli* cells exposed to 10 μg/mL MSNs. (TIF)

S3 Fig Pathway analysis of perturbed metabolites in *E*. *coli* cells exposed to 100 μg/mL MSNs. (TIF)

S1 Table Energy dispersive X-ray spectrometry (EDS) analysis of the MSNs. (DOCX)
