# Supplementary figures and images for: Investigating the Influence of MoS2 Nanosheets on E. coli from Metabolomics Level
Source: PLoS One. 2016 Dec 1;11(12):e0167245. doi: 10.1371/journal.pone.0167245 (PMC5132170; doi:10.1371/journal.pone.0167245)

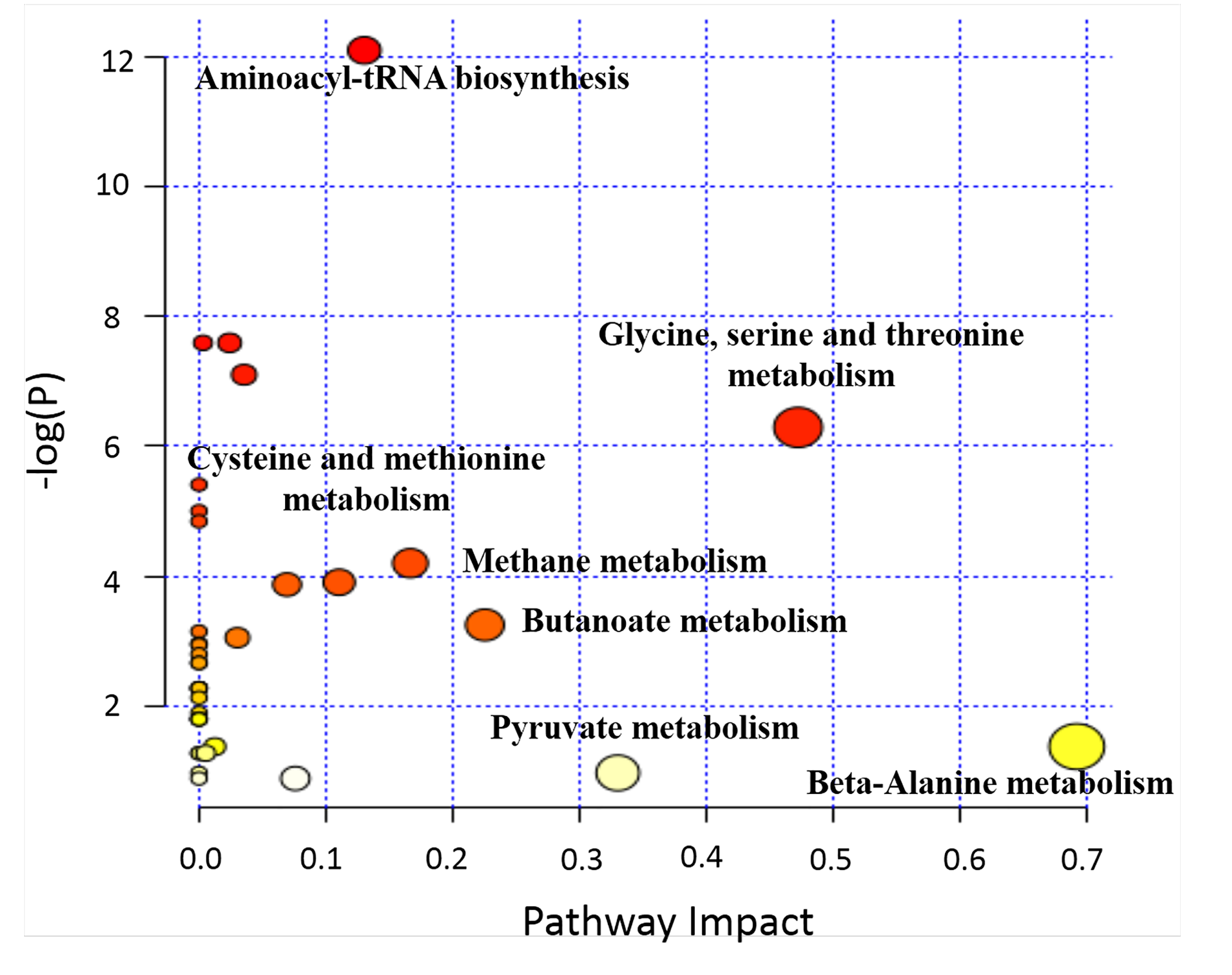

Supplement: S1 Fig — (TIF) [file pone.0167245.s001.tif]

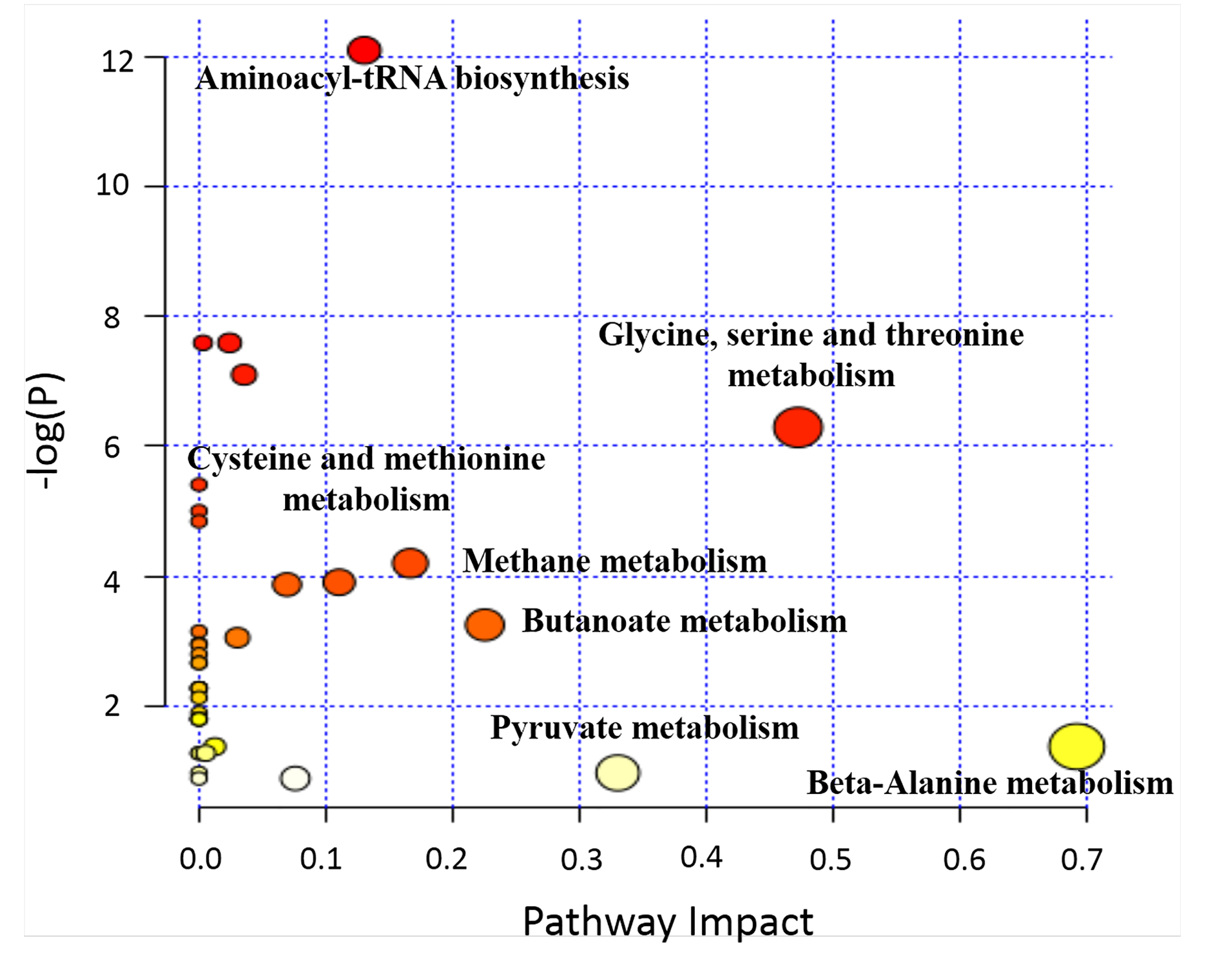

Supplement: S2 Fig — (TIF) [file pone.0167245.s002.tif]

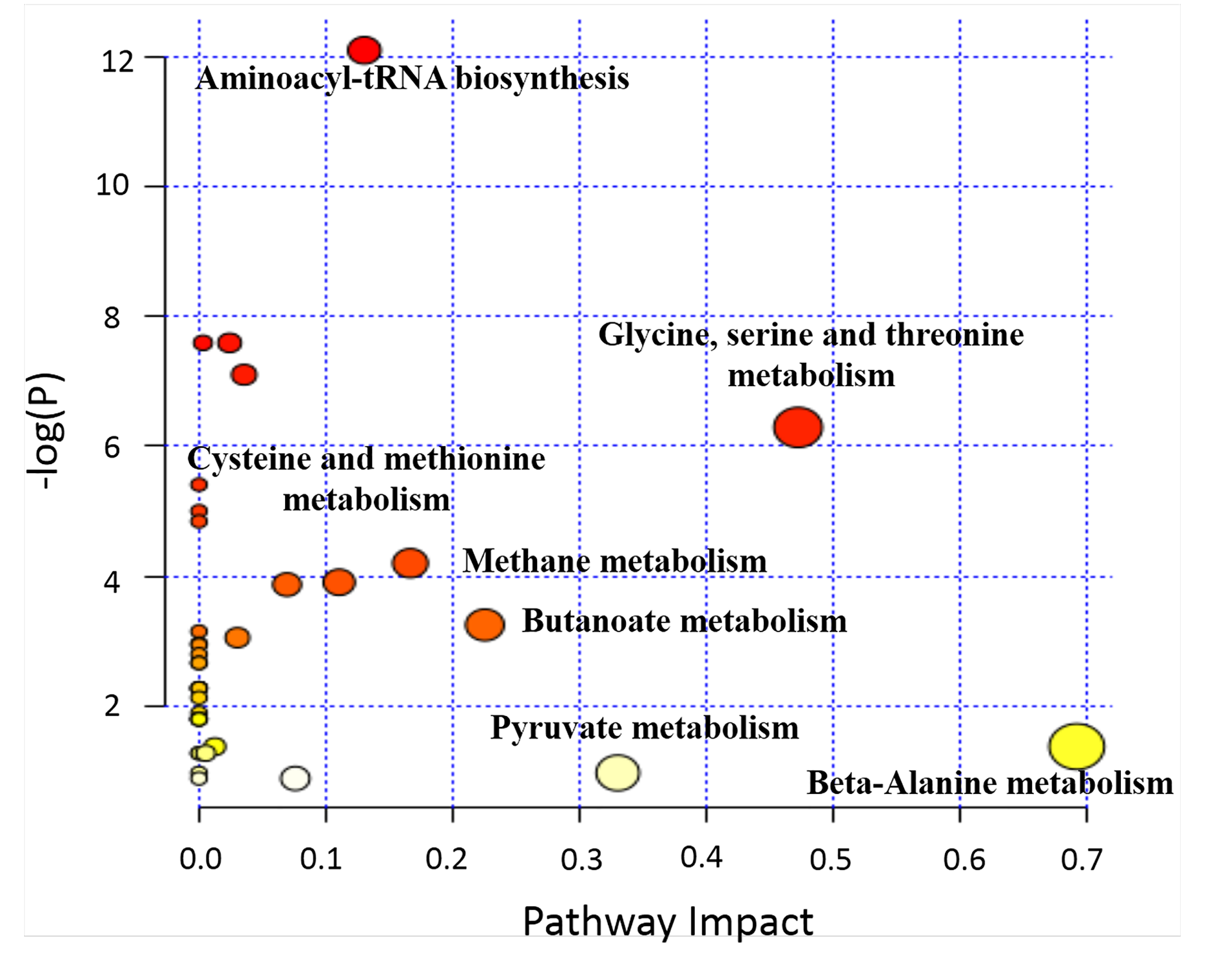

Supplement: S3 Fig — (TIF) [file pone.0167245.s003.tif]
