# Supplementary material for: Investigating the Influence of MoS2 Nanosheets on E. coli from Metabolomics Level
Source: PLoS One. 2016 Dec 1;11(12):e0167245. doi: 10.1371/journal.pone.0167245 (PMC5132170; doi:10.1371/journal.pone.0167245)
Supplement: S1 Table — (DOCX) [file pone.0167245.s004.docx]

| ***Element***  ***Line*** | ***Weight %*** | ***Atom %*** | ***Formula*** | ***Compnd %*** | ***Norm.***  ***Compnd%*** |
| --- | --- | --- | --- | --- | --- |
| ***S K*** | 38.05 | 64.76 | S | 38.05 | 38.05 |
| ***Mo L*** | 61.95 | 35.24 | Mo | 61.95 | 61.95 |
| ***Total*** | 100.00 | 100.00 |  | 100.00 | 100.00 |
